# Using a Clinically Interpretable End Point Composed of Multiple Outcomes to Evaluate Totality of Treatment Effect in Comparative Oncology Studies

**DOI:** 10.1001/jamanetworkopen.2023.19055

**Published:** 2023-06-21

**Authors:** Xiaofei Wang, Zachary R. McCaw, Lu Tian, Thomas E. Stinchcombe, Everett Vokes, Ethan B. Ludmir, Lee-Jen Wei

**Affiliations:** 1 Department of Biostatistics & Bioinformatics, Duke University Medical Center, Durham, North Carolina; 2 Insitro, South San Francisco, California; 3 Department of Biomedical Data Science, Stanford University, Stanford, California; 4 Duke Cancer Institute, Duke University Medical Center, Durham, North Carolina; 5 University of Chicago Comprehensive Cancer Center, Chicago, Illinois; 6 Department of Gastrointestinal Radiation Oncology, The University of Texas MD Anderson Cancer Center, Houston; 7 Department of Biostatistics, Harvard T.H. Chan School of Public Health, Boston, Massachusetts

## Abstract

This cohort study demonstrates how to use cumulative event count curves to create a clinically meaningful end point by simultaneously considering recurrence, progression, and survival times from the individual patient.

## Introduction

In oncology trials, disease progression, recurrence, and survival times routinely serve as efficacy measures for assessing treatment effect. The conventional method for quantifying the effect is to report 2 summary measures such as hazard ratios (HRs) by conducting separate analyses: an analysis for progression-free survival and another for overall survival. This procedure ignores the association of the occurrence of disease progression with death for the individual patient and does not provide an overall clinically meaningful and statistically efficient evaluation of the treatment effect.

As an example, a randomized clinical trial (RCT)^1^ was conducted by Cancer and Leukemia Group B (CALGB 9633) to investigate whether adjuvant paclitaxel and carboplatin would benefit patients with stage 1B non–small cell lung cancer. The HRs for disease-free survival and overall survival were 0.80 (95% CI, 0.62-1.021; *P* = .07) and 0.83 (95% CI, 0.64-1.08; *P* = .13), respectively.^1^ Neither analysis was statistically significant. To obtain an overall assessment of treatment benefit, one may combine the HRs, but the resulting summary is not clinically interpretable.^2^ Here, we show how to create a clinically meaningful end point by simultaneously considering recurrence, progression, and survival times from the individual patient.

## Methods

**Figure 1.**
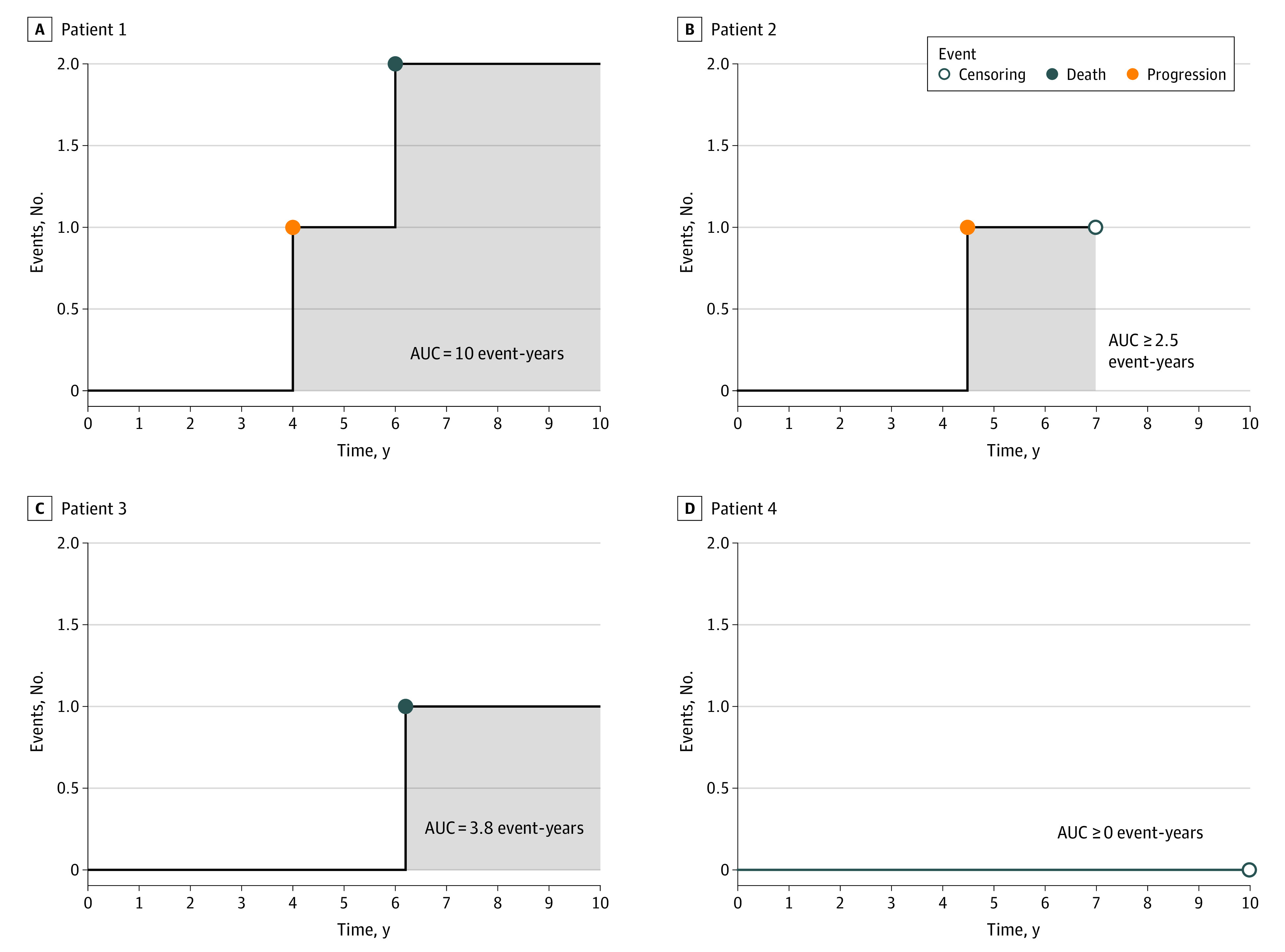
Example Temporal Profiles for Progression, Recurrence, and Death With Disease Burden Over a Study Period Graphs show examples of typical, per-patient cumulative event count curves for 4 hypothetical patients. AUC indicates area under the curve.

This cohort study follows the STROBE reporting guideline, and the analytic method used in this article is based on previous work by Claggett et al.^3^ Figure 1 displays examples of typical per-patient cumulative event count curves of 4 hypothetical patients. A patient potentially has 2 events over a study period: progression and/or death. The area under the curve (AUC) can be calculated to determine a patient’s total disease impact and burden over a study period and provide a clinically interpretable end point. For the first patient, the curve has 2 1-unit jumps reflecting progression and death. After progression at year 4, with 10-year follow-up, the patient lost 6 years (10 minus 4) of progression-free time and another 4 years due to death. The total time lost is 10 years, which is the AUC. With absent censoring (patients 1 and 3), the mean cumulative count curve for a group of patients and its AUC can be calculated by simple averaging. Standard methods are available for obtaining such a curve in the presence of censoring (patients 2 and 4).^3,4,5,6^ These methods were then used to create mean cumulative event count curves for the chemotherapy and control groups of the CALGB 9633 cohort.^1^

## Results

**Figure 2.**
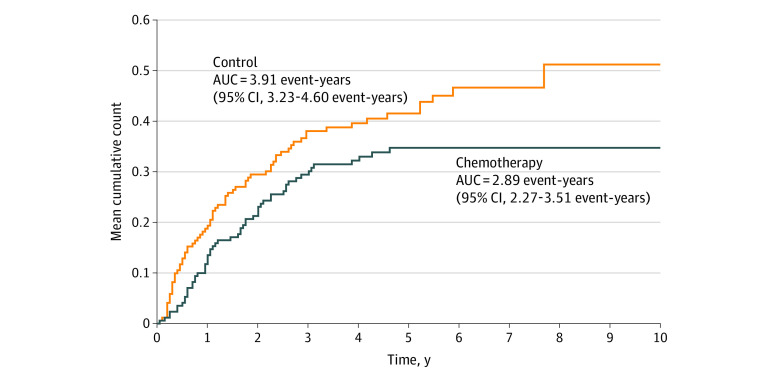
Mean Cumulative Count Curves for Disease Recurrence and Death Data are from the CALGB 9633 trial.^1^ AUC indicates area under the curve.

The patients’ baseline characteristics for CALGB 9633 were summarized in Strauss et al.^1^ Figure 2 displays the mean cumulative count curves for the chemotherapy and control groups. The curve from the chemotherapy group is uniformly lower than that from the control group; the AUCs are 2.89 and 3.91 event-years, respectively. The ratio of AUCs (chemotherapy vs control) is 74% (95% CI, 55%-96%; *P* = .02).^3^ The interpretation is that chemotherapy reduced the overall disease burden by 26%, on average, over 10 years. Contrary to HRs, AUC analysis yielded a significant and interpretable treatment benefit, supporting the usage of paclitaxel and carboplatin in the indicated population.

## Discussion

Analyses that use multiple measures to assess treatment outcomes have recently been implemented for clinical studies of various diseases,^3^ but not in cancer. As observed in this cohort study, the proposed method is statistically more powerful than considering outcomes separately. The proposed end point may include all relevant undesirable risk and benefit outcomes, such as the timing of premature treatment or study discontinuation due to toxic effects or lack of efficacy.^3,6^ Considering multiple measures to create a patient-level end point for evaluating the study therapy mirrors the clinical practice of managing a patient’s treatments. In practice, the truncation time should be prespecified. In this study, the results were robust, that is, the treatment difference remained significant for various truncation times. Note that the jump sizes for progression and death in Figure 1 are equal. A limitation of this study is that one may assign larger jumps to death, reflecting the varying degrees of severity of the different types of events, via approaches to quantifying the clinical utility of different disease states in the decision science. However, it is challenging in practice to get consensus among all stakeholders for the appropriate weights of different event types.

## Notes

Supplement.
Data Sharing Statement
