# Supplementary material for: Using a Clinically Interpretable End Point Composed of Multiple Outcomes to Evaluate Totality of Treatment Effect in Comparative Oncology Studies
Source: JAMA Netw Open. 2023 Jun 21;6(6):e2319055. doi: 10.1001/jamanetworkopen.2023.19055 (PMC10285578; doi:10.1001/jamanetworkopen.2023.19055)
Supplement: Supplement. — Data Sharing Statement [file jamanetwopen-e2319055-s001.pdf]

Data Sharing Statement

Wang. Using a Clinically Interpretable End Point Composed of Multiple Outcomes to Evaluate Totality of Treatment Effect in Comparative Oncology Studies. *JAMA Netw Open*. Published June 21, 2023. doi:10.1001/jamanetworkopen.2023.19055

Data
Data available: No

Additional Information
Explanation for why data not available: Data from de-identified patients from randomized clinical trials were used to illustrate the new method. The authors have direct access to the data file. Data available on request due to privacy/ethical restrictions.
